# Nuclear DNA Amounts in Chinese Bryophytes Estimated by Flow Cytometry: Variation Patterns and Biological Significances

**DOI:** 10.3390/plants12071564

**Published:** 2023-04-05

**Authors:** Dandan Li, Guangyu Luo, Shuiliang Guo, Ruoling Huang, Jun Yang, Tong Cao, Jing Yu

**Affiliations:** College of Life Sciences, Shanghai Normal University, Shanghai 200234, China

**Keywords:** bryophyte, China, nuclear DNA amount, flow cytometry, variation

## Abstract

There exists an obvious gap in our knowledge of the nuclear DNA amount of bryophytes, not only in terms of the low number of species represented, but also in systematic and geographic representation. In order to increase our knowledge of nuclear DNA amounts and variation patterns in bryophytes, and their potential phylogenetic significances and influences on phenotypes, we used flow cytometry to determine the DNA 1C values of 209 bryophyte accessions, which belong to 145 mosses and 18 liverworts collected from China, by using *Physcomitrella patens* as a standard. We quantified the differences in DNA 1C values among different orders and families and constructed a phylogenetic tree of 112 mosses with four gene sequences (*nad*5, *rbc*L, *trn*L-F, and 18S-ITS1-5.8S-ITS2-26S). DNA 1C values were mapped onto the phylogenetic tree to test a potential phylogenetic signal. We also evaluated the correlations of the DNA 1C value with the sizes of individuals, leaves, cells, and spores by using a phylogenetically controlled analysis. New estimates of nuclear DNA amounts were reported for 145 species. The DNA 1C values of 209 bryophyte accessions ranged from 0.422 pg to 0.860 pg, with an average value of 0.561 pg, and a 2.04-fold variation covered the extremes of all the accessions. Although the values are not significantly different (*p* = 0.355) between mosses (0.528 pg) and liverworts (0.542 pg), there are variations to varying extents between some families and orders. The DNA 1C value size exerts a positive effect on the sizes of plants, leaves, and cells, but a negative effect on spore size. A weak phylogenetic signal is detected across most moss species. Phylogenetic signals are comparatively strong for some lineages. Our findings show that bryophytes have very small and highly constrained nuclear DNA amounts. There are nucleotype effects of nuclear DNA amounts for bryophytes at the individual, organ, and cell levels. We speculate that smaller nuclear DNA amounts are advantageous for bryophytes in dry environments. Significant differences in the DNA 1C values among some moss families and orders, as well as phylogenetic signals for some lineages, imply that nuclear DNA amount evolution in mosses seems to be unidirectional.

## 1. Introduction

The amount of DNA in the whole chromosome complement irrespective of the ploidy level of the organism is referred to as the DNA C-value, while the amount of nuclear DNA in an unreplicated haploid chromosome complement is referred to as the genome size (DNA 1C value) [1,2]. Nuclear DNA amount is an important biodiversity character with fundamental biological significance [3,4], and remains vital for many diverse fields of biology.

Since 1976, lists of DNA amounts in different plant categories, complied for reference purposes, have been published [5], and the data have been pooled and released in a database since 1997. Nuclear DNA amount data for 12,273 species, comprising 10,770 angiosperms, 421 gymnosperms, 303 pteridophytes, 334 bryophytes (212 mosses, 109 liverworts, and 13 hornworts), and 445 algae, were included in the updated Version 4.0 of the Plant DNA C-value Database [2]. These lists and databases have been widely used for comparative studies [6,7,8,9]. However, the availability of nuclear DNA amount data varied widely among different plant categories [2]. Large gaps still exist in our knowledge of nuclear DNA amounts. Nuclear DNA amount data in many species and geographic regions have not yet been reported. Improving geographic and taxonomic representations were the two main targets in the following collection of nuclear DNA amount data [10,11].

DNA C-values in land plants (comprising bryophytes, lycophytes, monilophytes, gymnosperms, and angiosperms) vary ca. 1000-fold from approx. 0.11 to 127.4 pg [7]. The values highly vary among different angiosperm families; the average DNA 1C-vlaue differs 60-fold, from 0.62 pg (Salicaeae) to 26.58 pg (Alstroemeriaceae); nuclear DNA amounts are significantly lower in dicots than in monocots and are also significantly lower in non-perennials than in perennials [12]. However, the patterns of variation in nuclear DNA amounts among different bryophyte taxa are poorly understood.

Bryophytes are next to angiosperms in species number. Despite the numerous nuclear DNA amount data that exist in seed plants, the Plant DNA C-value Database revealed an obvious gap in our knowledge of the nuclear DNA amounts of bryophytes, not only in terms of the low number of species represented, but also in terms of systematic and geographic representation [2]. According to the updated DNA C-value Database (version 4.0), genome sizes (DNA 1C values) of 334 bryophyte species have been determined so far, mainly reported by Voglmayr (137 species) [13], Temsch et al. (77 species) [14], Bainard et al. (56 species) [10], Bainard (32 species) [15], Bainard and Villarreal (23 species) [16], and Greihuber et al. (5 species) [17]. Recently, the genome sizes of 33 moss species were reported by Bainard et al. [11]. Compared with those of angiosperms (approx. 4.2%), the nuclear DNA amount data of bryophyte species were relatively scarce (approx. 2.6% of ca. 12,800 bryophyte species, Goffinet and Shaw [18]). Concerning nuclear DNA amount data, taxonomic representation is also problematic. So far, no nuclear DNA amount estimate is available for more than 52% of bryophyte families. Additionally, although there is evidence of nuclear DNA amount data in some families, the proportion of their species with available nuclear DNA amount data was also rather low. For example, only 4 of the 447 species in the family Fissidentaceae, or less than 1% of the family’s species, have been determined for their nuclear DNA amounts [2]. Despite the rich species of bryophytes in China, no nuclear DNA amount data were available for bryophyte accessions collected from China up until now.

Although previous studies revealed that genome size variation exhibits phylogenetic signals for some liverworts [10], mosses [11], and hornworts [16], more data were still needed to improve the systematic and geographic representation of the phylogenetic signal in bryophytes. Because of their dominant gametophytes, lack of vascular tissues, and poikilohydric strategy, bryophytes are unique among land plants [19]. Furthermore, bryophytes are characterized by their small size, high sensitivity to habitats and substrate specificity, and a short generation time, as well as their fast colonization–extinction rate [20,21]. However, there have been few studies on the relationship between bryophyte nuclear DNA amounts and morphological traits.

The objectives of the present work were to (1) increase our knowledge of nuclear DNA amounts and their variation patterns in bryophytes; (2) clarify whether nuclear DNA amounts exert potential nucleotype effects on the phenotypes of bryophytes, and their possible ecological significances; and (3) further confirm whether phylogenetic signals exist for nuclear DNA amounts in bryophytes.

## 2. Results

### 2.1. General Aspects

The DNA 1C values of 209 bryophyte accessions were determined. These accessions included 145 mosses (belonging to 86 genera and 34 families) and 18 liverworts (belonging to 13 genera and 12 families). New DNA 1C values for 9 families, 64 genera, and 145 species were reported (Appendix A).

### 2.2. Variation Pattern of Nuclear DNA Amounts

The average DNA 1C value of the 209 accessions was 0.561 pg. Among the 163 species, *R. giganteum* with a DNA 1C value of 0.862 pg was ranked the largest, followed by *Leucobryum scabrum* Sande Lac. (0.846 pg), *Rhytidiadelphus triquetrus* (Hedw.) Warnst. (0.81 pg), *Lescuraea radicosa* (Mitt.) Mönk. (0.788 pg), *Polytrichum juniperinum* Hedw. (0.76 pg), and *Climacium dendroides* (Hedw.) F. Weber & D. Mohr (0.737 pg). *Macromitrium japonicum* Dozy & Molk. with 0.422 pg ranked the smallest, followed by *Bartramia ithyphylla* Brid. (0.434 pg), *Schistidium striatum* Brid. (0.442 pg), *Leucobryum glaucum* (Hedw.) Ångstr. (0.458 pg), *Hookeria acutifolia* Hook. & Grev. (0.458 pg), and *Homomallium connexum* (Cardot) Broth. (0.458 pg). Among 209 accessions, 2.04-fold variation covers the extremes of the whole sample. The values were not significantly different (*p* = 0.355) between mosses (0.528 pg) and liverworts (0.542 pg) (Appendix A).

**Figure 1:**
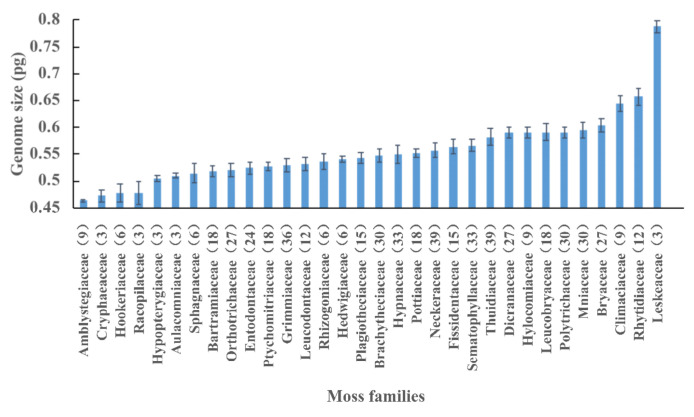
Mean values and standard errors (bars) of DNA 1C values for 32 moss families. Sample number is listed in parentheses.

**Figure 2:**
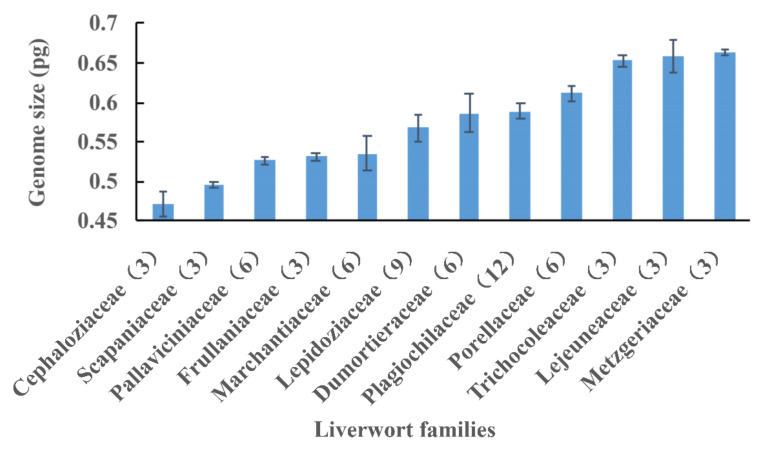
Mean values and standard errors (bars) of DNA 1C values for 12 liverwort families. Sample number is listed in parentheses.

Among the 34 moss families, Leskeaceae, with an average DNA 1C value of 0.788 pg, was ranked the highest, and Amblystegiaceae, with 0.463 pg, ranked the lowest. The variation degree of DNA 1C values within a family highly varied among 32 moss families. According to the standard errors, Amblystegiaceae, Hedwigiaceae, Aulacomniaceae, Hypopterygiaceae, Pottiaceae, and Ptychomitriaceae had very constrained DNA 1C values, while others, such as Racopilaceae, Sphagnaceae, Hookeriaceae, Hypnaceae, and Leucobryaceae, had more variation in their DNA 1C values (Figure 1). For liverwort families, Dumortieraceae, Marchantiaceae and Lejeuneaceae had more variable DNA 1C values, while Metzgeriaceae, Scapaniaceae, Frullaniaceae and Pallaviciniaceae had comparatively constrained DNA 1C values (Figure 2).

Among the major orders, Bryales (0.602 ± 0.022 pg) had the largest DNA 1C value, which was significantly larger (*p* < 0.05) than Hypnales (0.564 ± 0.007 pg), Grimmiales (0.529 ± 0.010 pg), Orthotrichales (0.521 ± 0.021 pg) and Bartramiales (0.520 ± 0.020 pg), and slightly larger (*p* < 0.2) than Pottiales (0.551 ± 0.026 pg).

Among the 163 bryophyte species, 41 were collected from more than one locality. Variations in DNA 1C values to varying extents between geographic accessions were detected for 18 species (*p* < 0.01, accounting for 43.9%), 8 species (*p* < 0.05, 19.51%), and 3 species (*p* < 0.1, 7.32%).

### 2.3. Relationships between DNA 1C Values and Morphological Traits

**Figure 3:**
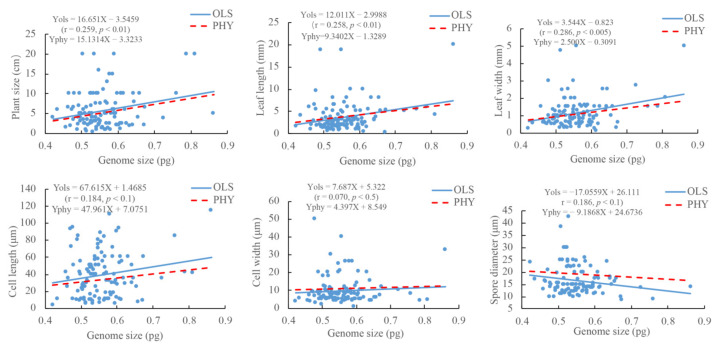
Relationships of DNA 1C value (genome size) with the sizes of plants, leaves, cells and spores for the 112 moss species. Note: the OLS line represents the relationship modeled by using the ordinary least squares, while the PHY line represents the relationship by using phylogenetically controlled analysis.

The DNA 1C values of the 112 mosses were positively related to plant sizes (*p* < 0.01), leaf length (*p* < 0.01) and width (*p* < 0.005), cell length (*p* < 0.1) and width (*p* < 0.5), but negatively related to spore sizes (*p* < 0.1). The above relationships also existed after taking into account phylogenetic non-independence (Figure 3).

### 2.4. Phylogenetic Signal for DNA 1C Value among Moss Taxa

**Figure 4:**
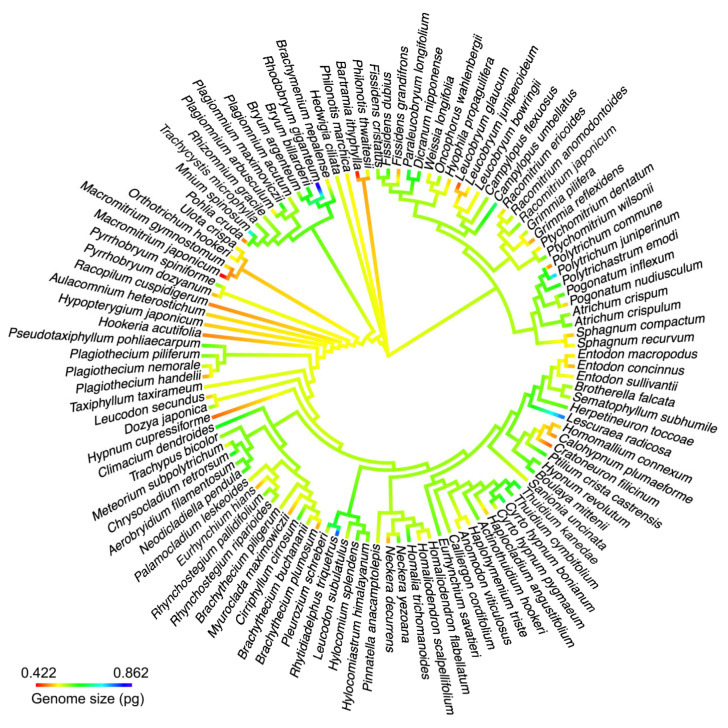
Continuous character state mapping of the average DNA 1C values (genome size), ranging from 0.422 pg (red) to 0.862 pg (blue), onto a ultrametric tree that includes 112 moss species, using the ContMap function with default settings from the phytools package in R.

**Figure 5:**
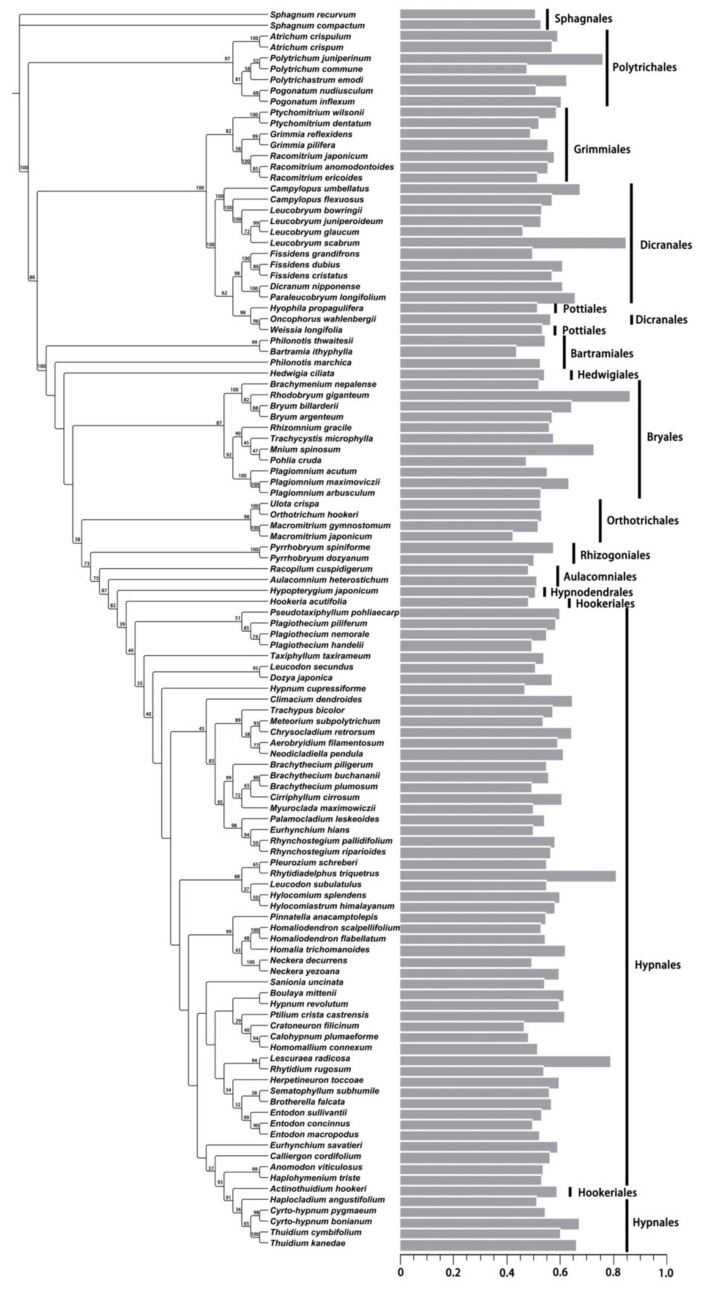
Phylogram of 112 moss species with the average DNA 1C value for each species plotted and the moss orders indicated with vertical bars. The maximum likelihood bootstrap values are labelled above the branches.

**Figure 6:**
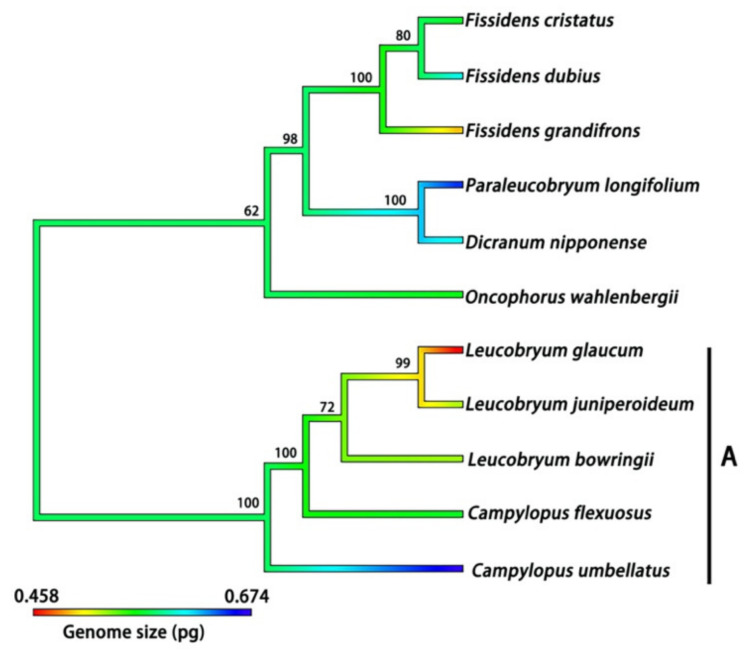
Continuous character state mapping of the average DNA 1C values (genome sizes), ranging from 0.458 pg (red) to 0.674 pg (blue), onto an ultrametric tree that includes 11 species of the order Dicranales by using the ContMap function with default settings from the phytools package in R. Clade A indicated a strong phylogenetic signal of DNA 1C value across the five species. The maximum likelihood bootstrap values are labelled above the branches.

**Figure 7:**
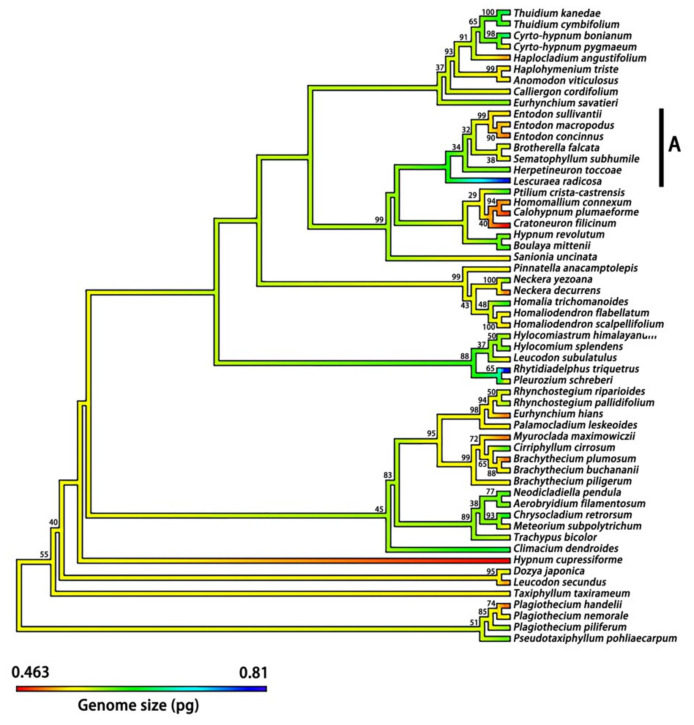
Continuous character state mapping of the average DNA 1C values (genome sizes), ranging from 0.463 pg (red) to 0.810 pg (blue), onto a ultrametric tree that includes 57 species of the order Hypnales by using the ContMap function with default settings from the phytools package in R. Clade A indicated a strong phylogenetic signal of DNA 1C value across the seven species. The maximum likelihood bootstrap values are labelled above the branches.

The phylogenetic tree included 112 moss species, accounting for 78.32% of the total moss species whose DNA 1C values were determined in the present study (Figure 4 and Figure 5). Average DNA 1C values for these species ranged from 0.422 to 0.862 pg. The K-statistic for the whole tree with 112 moss species was 0.120, with a *p*-value of 0.203, indicating a weak phylogenetic signal for DNA 1C values across the tree. The phylogenetic signal was comparatively strong for some lineages, such as Dicranales (K-statistic = 0.488, *p* < 0.20) and its Clade A (K-statistic = 1.169, *p* < 0.05) (Figure 6), and Clade A of Hypnales (K-statistic = 2.429, *p* < 0.001) (Figure 7).

## 3. Discussion

The studies relevant to nuclear DNA amounts have been shown to be useful in elucidating taxonomic, evolutionary, and ecological problems in a number of plant taxa [9,12,22,23,24,25,26]. Therefore, there is a continuing need to obtain more data on DNA 1C values for plant taxa.

### 3.1. Credibility of the Nuclear DNA Amounts We Determined

Our result of the moss accessions is mostly consistent with those of some previous reports. For example, a mean DNA 1C value of 0.52 pg for 209 moss accessions was recorded in the Bryophyte DNA C-value Database [2]. Voglmayr [13] reported a mean value of 0.509 pg for 137 moss species, Temsch et al. [27] reported a minimum of 0.39 pg and a maximum of 0.94 pg, and mean of 0.530 pg for 30 moss species, Greihuber et al. [17] reported a minimum of 0.44 pg, and maximum of 0.95 pg and a mean of 0.646 pg for five moss species, and Renzaglia, Rasch and Pike [28] reported an interspecific variation in DNA 1C values from 0.38 pg to 0.92 pg, with a mean of 0.544 pg for nine mosses by measuring their sperms using flow cytometry. Recently, Bainard et al. [11] reported DNA 1C values of mosses ranging from 0.25 pg (*Dicranoweisia cirrata* (Hedw.) Lindb. ex Milde) to 1.18 pg (*Leucolepis acanthoneuros* (Schwägr.) Lindb.). The mean DNA 1C value of the liverworts in the present work is smaller than the data reported by Temsch et al. (0.736 pg for 43 liverworts) [14] and Bainard et al. (0.853 pg for 67 liverworts) [10]. The lower mean value of DNA 1C values for liverworts in this study was probably attributed to the insufficient liverwort samples in our work.

The DNA 1C value range is much broader in Voglmayr’s paper, ranging from 0.174 to 2.16 pg, while it is much narrower in the present work, ranging from 0.422 pg to 0.860 pg. This is possibly due to different samples and determining methods. In fact, among the data of 289 accessions determined by Voglmayr [13] (determined by flow cytometer or by Feulgen densitometry), only 14 accessions each had a DNA 1C value larger than 1.0 pg and about 95% of the accessions had DNA 1C values smaller than 1.0 pg. In the DNA C-value database [2], among the 165 moss accessions determined by flow cytometry, only 7 accessions have a DNA 1C value larger than 1.0 pg, while about 96% of the accessions have a DNA 1C value smaller than 1.0 pg. Additionally, the data in the Kew database and Voglamyr‘s report were determined by flow cytometry or by Feulgen densitometry. According to our analyses of the data from the Kew database (version 4.0), the average 1C value (pg) determined by flow cytometry is 4.68 pg for 7626 angiosperm species, while that by Feulgen densitometry is 6.58 pg for 2931 angiosperm species. The case is the same for mosses. The average DNA 1C values determined by Feulgen densitometry (37 accessions) are 0.548 pg, while those determined by flow cytometry (165 accessions) are 0.509 pg. If the five largest data are excluded, the average DNA 1C values obtained by flow cytometry (160 accessions) are only 0.474 pg. Therefore, the data determined by flow cytometry are essentially smaller than those determined by Feulgen densitometry. The phenomenon that the data in the present work are generally smaller than those previously reported is possibly due to the fact that we used flow cytometry to determine the DNA 1C-values.

### 3.2. Variation Patterns of the Nuclear DNA Amounts in Bryophytes

The mean DNA 1C value of all the accessions was 0.53 pg, which was lower than that of 334 accessions (0.916 pg, DNA 1C value) in the Bryophyte DNA C-value Database [2]. Considering the high proportion of mosses in our collections, the result is reasonable and acceptable. Among the 209 accessions determined here, 188 are moss accessions; their DNA 1C values varied from 0.442 pg to 0.862 pg, with a mean value of 0.529 pg. The DNA 1C values of the mosses in the DNA C-value Database varied from 0.17 to 2.05 pg, with a mean of 0.519 pg [2].

Relatively weak interspecific variation in the DNA 1C value was detected for our accessions. In addition, 2.16-fold interspecific variation covers the extremes of the accessions, with an average value of 0.53 pg, a maximum of 0.862 pg in *Rhodobryum giganteum*, and a minimum of 0.398 pg in *Macromitrium japonicum*. This variation is much low compared with the 127.9-fold interspecific variation recorded in the Bryophyte DNA C-value Database [2]. Among the three groups of bryophytes in the database, liverworts have the largest DNA 1C value (a mean of 1.89 pg) and the largest interspecific variation (102 species, 97.43-fold), followed by mosses (a mean of 0.52 pg and an interspecific variation of 12.06-fold for 209 species), and hornworts have the smallest DNA 1C value (a mean of 0.249 pg) and interspecific variation (4.56-fold for 23 species). The 209 accessions did not contain hornworts and contained only 20 liverwort samples (accounting for less than 10% of the total accessions). Our result is mostly consistent with that of Voglmayr [13], who reported a two-fold interspecific variation in DNA 1C values from 0.3 to 0.6 pg for the majority of species. Although there existed a ca. 12-fold interspecific variation in DNA 1C values within mosses, 80% of the values were restricted to a range between 0.25 and 0.6 pg (2.4-fold variation) [13].

Among the ten moss orders we examined, the Bryales have the largest DNA 1C value (0.602 ± 0.022 pg), which is consistent with the speculation by Bainard et al. [11]. Considering significant differences in the DNA 1C value existed among some families, such as Leucobryaceae, Rhytidiaceae, Entodontaceae, Orthotrichaceae, Dicranaceae, Mniaceae, Bartramiaceae and Grimmiaceae (Table S1), the DNA 1C value evolution in mosses seems to be unidirectional.

*Hookeria lucens* was reported to have a relatively large DNA 1C value (1.61 pg) by Bainard et al. [11], but our investigation showed that *Hookeria acutifolia* Hook. & Grev. has a relatively small DNA 1C value (0.478 ± 0.017 pg). Therefore, more samples were needed to clarify the DNA 1C value throughout the order.

### 3.3. Nucleotype Effects and Possible Ecological Significance of Nuclear DNA Amounts in Bryophytes

Nuclear DNA content could affect the phenotype through the biophysical effects of its mass and volume, with the latter defined as nucleotype effects [29]. Nucleotype variation in nuclear DNA amount sets absolute limits on the minimum size and mass of cells. Such effects are additive in complex multicellular vascular plants, and the potential effects of the DNA 1C value can apply to cells, organs, and organisms, and act on many aspects of the life history of the plant [30]. In angiosperms, nuclear DNA amount positively correlated with the volume and weight of chromosomes [31,32,33], nuclear and cell sizes [34], epidermal cell size and leaf size of *Lolium perenne* L. [35], leaf width in the species of *Nerine* (Amaryllidaceae) [36], and plant height in *Sencio* in Australia [29]. However, the nucleotype effects of nuclear DNA content on phenotype have received little attention in bryophytes. Here, we detected significantly positive correlations of the DNA 1C value with plant size, leaf size, and cell size (Figure 3), confirming the nucleotype effects of nuclear DNA content on the phenotype of bryophytes. The nucleotype effects of the DNA 1C value on the phenotype of bryophytes appeared at the individual, organ, and cell levels.

Early studies showed that the nuclear DNA amount negatively correlated with the duration of the mitotic and meiosis cycle [37,38], and minimum generation time [39]. Cutler et al. [40] suggested that smaller cells help plants to resist moisture stress because they maintain turgor with solute accumulation under lower water potential values compared to larger cells. Small cells often have small nuclear DNA amounts. According to Rejmánek [41], a low nuclear DNA content seems to be a result of selection for short minimum generation times in extreme cold environments. We speculate that a small nuclear DNA amount is also advantageous for bryophytes in dry environments. Bryophytes are generally sensitive to dry environments [21,42]. A small nuclear DNA amount allowed bryophytes to rapidly develop in a time-limited duration of favorable moisture availability in dry regions by a rapid mitotic cycle and a short duration of meiosis.

The spores of bryophytes are somewhat similar to the pollens of angiosperms as reproduction units. In angiosperms, significantly positive correlations of nuclear DNA content with pollen size were reported in *Armeria maritima* (Mill.) Willd. (Plumbaginaceae) [43], and in some cereal species [39]. However, we detected a negative relationship between spore size and DNA 1C value in 112 moss species (Figure 3). Löbel and Rydin [44] suggested that species with larger spores have a higher probability surviving in harsher habitats (e.g., dry habitats). Therefore, regardless of the fact that a small DNA 1C value allowed bryophytes to rapidly develop in a time-limited duration of favorable moisture availability in dry regions, another advantage for species with a smaller DNA 1C value and larger spores may be that larger spores have a higher probability to survive in a dry habitat. Proctor et al. [45], Baniaga et al. [26] and Bainard et al. [11] thought that desiccation tolerance might be an important selective pressure for plants to keep a relatively small DNA 1C value, which was consistent with our results. Nevertheless, more data will need to be collected before the relationship of spore size with the DNA 1C value in mosses can be clearly established.

To further rigorously clarify the relationships between nuclear DNA amounts and ecological adaptation for mosses, we must collect the global geographic distribution data of the mosses with known nuclear DNA amounts, and the corresponding climate data of these distribution points, then quantify the relationships among the climates, nuclear DNA amounts, and morphological traits. This will possibly allow us to better understand the ecological significances of the nuclear DNA amounts in mosses.

### 3.4. Phylogenetic Signals of Nuclear DNA Amounts in Bryophytes

According to Leitch et al. [2], the DNA 1C value is much smaller (a mean value of 0.52 pg) in mosses than in angiosperms (a mean of 5.13 pg), and the interspecific variation is much weaker in mosses (12.04-fold for most species) than in angiosperms (ca. 2000-fold). What is the reason that the DNA 1C values are so small and constant in mosses? DNA 1C value variation is likely to be a whole-organism phenomenon that can be studied at the developmental and ecological levels [46]. Renzaglia et al. [28] thought that the selection of biflagellated sperm may have favored a low nuclear DNA amount.

The variations in DNA amount have been found to be linked with phylogenetic signals across land plants [7], flowering plants [47,48] and a number of angiosperm taxa, such as *Allium* [49], *Capsicum* (Solanaceae) [50], Poaceae [51], and Bromelioideae of Bromeliaceae [52]. Bainard and Villarreal [16] reported a 20.46-fold interspecific variation in the DNA 1C-value from 0.27 to 20.46 pg for 67 hornwort species from 33 families using flow cytometry and detected a strong phylogenetic signal of DNA 1C-value across the liverwort phylogeny. Recently, Bainard et al. [11] detected a phylogenetic signal of DNA 1C values across the phylogeny of mosses based on the data they determined and those from previous studies. In our moss sampling, there existed only a two-fold interspecific variation in the DNA C-value from 0.422 to 0.862 pg and a weak phylogenetic signal across the phylogenetic tree, which we produced based on four gene regions available in the NCBI database. However, for some lineages of Dicranales and Hypnales, the phylogenetic signal was comparatively strong, and the variation in the DNA 1C value was roughly correlated with their phylogenetic relatedness (Figure 6 and Figure 7). The above results were consistent with that of Bainard et al. [11]. The very small range of DNA 1C values across the 145 mosses we examined likely indicated that the DNA 1C values remain constrained in mosses and there has not been much divergence in the DNA 1C values over evolutionary history, which is consistent with the result of Baniaga et al. [26] that small nuclear genomes of *Selaginella* were associated with a low rate of DNA 1C value evolution. Additionally, many new DNA 1C value estimates reported for 145 bryophyte species are valuable for a better understanding of the phylogenetic signal of DNA 1C values across the phylogeny of the whole bryophyte group.

## 4. Materials and Methods

### 4.1. Materials

The plant materials are presented in Appendix A. Shuiliang Guo and Tong Cao identified voucher specimens, which were deposited at the bryophyte herbarium of Shanghai Normal University (SHTU). The taxonomy of species mainly follows the work of Jia and He [53].

### 4.2. Nuclei Isolation

The protocol for isolating nuclei was adapted from that of Johnston et al. [54]. Bryophyte tissues (5 to 15 moss fresh shoot tips and 1–2 cm^2^ fresh liverwort tissue, ca. 10 mg air-dried tissue) were washed to remove soil, chemicals, and other organisms that might react with the chemicals and alter the results. The tissue was chopped at room temperature, with a razor blade in about 0.55 mL of isolation buffer to homogenize the tissues and release the nuclei. The composition of the isolation buffer (200 mL) contained 45 mM MgCl_2_, 30 mM sodium citrate, 20 mM MOPS and 0.1% (*w*/*v*) Triton X−100 (reminding deionized water, pH 7.0) [55]. The nucleus suspension was then filtered (with a 10 mL syringe and a 30 μm nylon mesh) to remove debris that might block the flow cell. The nuclei suspension was filtered into a 1.5 mL tube to centrifuge at 1600 r/min for 5 min. After removal of the supernatant liquid, the nuclei were stained with 150 µg·mL^−1^ propidium iodide in the presence of 0.5 µg·mL^−1^ RNase. The mixture was dyed at 4 °C under the dark for 20 min. From each species, three accessions were randomly selected for DNA amount measurement [56].

### 4.3. Nuclear DNA Amount Measurement

*Physcomitrella patens* (Hedw.) Bruch & Schimp. was used as a standard (0.53 pg/DNA 1C value) [57] because the material of the species was widely available, quick and easy to grow, suitable for FCM protocols, and with an appropriate genome size for bryophytes [11]. Additionally, *Physcomitrella patens* was the first non-seed plant to have its genome sequenced, with verified genome size stability within well-delimited species [58]. The species has very few secondary compounds, which will interfere with quantitative DNA staining [59]. Therefore, *P. patens* has been used as a standard in measuring the genome size of seed plants [28] and bryophytes [60]. We used the gametophytes of the species, which were cultured in a growth chamber.

A flow cytometer (FACSCalibur, BD Bioscience, Mountain View, CA, USA) was used for the nuclei suspension analysis. The laser-emission wavelength was adjusted to 488 nm. Each sample consisted of 300 μL of nuclei suspension, and analysis was conducted at a data rate of 100–150 nuclei per second. The histogram was analyzed by using the ModFit LT software to obtain the G0/G1 peak (namely the fluorescence value), and the variation coefficient of the G0/G1 peak (CV% = standard deviation/mean ×100) [61]. If the CV value is less than 5%, the results are acceptable; otherwise, they are not [62].

To determine the DNA 1C value, the relative position of the bryophyte 1C peak was compared to the position of the standard 1C peak. The 1C peak was observed for the bryophyte as the haploid (gametophytic) tissue was analyzed. The nuclear DNA amount of the sample could be calculated as follows:

(1) $$\mathrm{Sample}\;1C\;\mathrm{value}\;\left(\mathrm{DNA}\;\mathrm{pg}\right)=\mathrm{Standard}\;1C\;\mathrm{value}\times \frac{\mathrm{sample}\;1C\;\mathrm{meak}\;\mathrm{peak}\;\mathrm{position}\;\mathrm{of}\;\mathrm{three}\;\mathrm{replicates}}{\mathrm{standard}\;1C\;\mathrm{mean}\;\mathrm{peak}\;\mathrm{position}\;\mathrm{of}\;\mathrm{three}\;\mathrm{replicates}}$$

**Figure 8:**
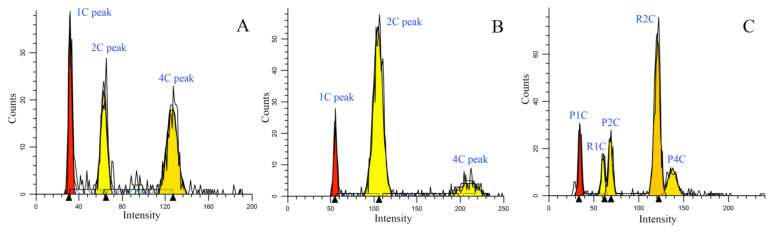
Histograms of counts versus fluorescence using flow cytometry for *Physcomitrella patens* (**A**), *Rhodobryum giganteum* (**B**), and for *R. giganteum* with *P. patens* as the internal standard (**C**). Note: In Figure C, P1C, P2C and P4C are peaks for *P. patens*, R1C and R2C for *R. giganteum,* respectively.

As is the case with most moss species, *Physcomitrella patens* is a typical endopolyploidy species [63] (Figure 8). If using the internal standard method, the 1C, 2C, and 4C peaks of the standard and the peaks of the sample are not easily separated and identified. Thus, we compared the genome size of *Rhodobryum giganteum* (Schwägr.) Paris determined by using *P. patens* as an internal standard with the value obtained by using *P. patens* as an external standard. We found that the genome size of *R. giganteum* obtained by using the external standard method was 0.862 ± 0.006, while that of the internal standard method was 0.860 ± 0.003 (with three replicates) (Figure 8), revealing that their difference was insignificant (*p* > 0.5). Therefore, the genome sizes were estimated in the present study by using *P. patens* as an external standard. Flow cytometry using an external standard was also used to measure the nuclear DNA amount in previous works [64,65,66,67,68,69,70,71]. To control the negative influences of the external standard method on the result as much as possible, the instrument settings were adjusted to control the “drift” in the peak location over time, and the histogram of the standard (*P. patens*) was obtained every time for comparison with that of the new species. Both the sample and the standard were measured with three replicates for each species.

### 4.4. Data Analysis

All the data were expressed as means ± standard errors with three replicates. One-way analysis of variance (ANOVA) was employed to test the differences among the taxa in their genome sizes using the procedures in the SPSS 22.0 statistical package (IBM Corp., Armonk, NY, USA). The least significant difference (LSD) method was employed.

To test whether the DNA 1C values are of evolutionary significance, we constructed a phylogenetic tree including 112 out of the 143 moss species whose genome sizes had been estimated. These species were selected because their four gene regions (*nad*5, *rbc*L, *trn*L-F; 18S-ITS1-5.8S-ITS2-26S) were available in the NCBI database (Table S2). We did not perform phylogenetic analyses of liverworts due to insufficient sampling.

Sequence chromatograms were compiled using Seqman II (DNASTAR Inc., Madison, WI, USA) and then automatically aligned in PhyDE 0.9971 [72]. Regions of partially incomplete data at the beginning and end of sequences were excluded from subsequent analyses. Gaps were treated as missing data. A total of 6106 base pairs, which included 3304 variable sites and 2477 parsimony-informative sites, were used to construct the phylogenetic tree.

We used MrModeltest v. 2.4 [73], which is incorporated in PAUP 4.0a168 [74], to select the best-fit nucleotide substitution model for each gene according to the corrected Akaike information criterion (AICc). The relevant parameters were set accordingly for each compartment. The phylogenetic tree was constructed using RAxML 8.2.10 [75]. The trees were visualized and annotated in TreeGraph 2 [76].

Using the ContMap function with default settings from the phytools package, DNA 1C values were mapped onto the phylogenetic tree. The K-statistic of the phylogenetic signal was calculated by using the phylosig function from the phytools package [77].

To detect the possible relationships between the DNA 1C value and morphological traits, data on plant size (at the individual level), leaf length and width (at the organ level), cell length and width, and spore diameter (at the cell level) were collected from relevant literature (Table S3). Plant size refers to the height of the main stem for acrocarpous mosses or the creeping stem for pleurocaropus mosses. Leaf length and width refer to those of branch leaves. Cell length and width refer to those of the median cells in branch leaves. The mean values of the above indices were used in relevant analyses.

The correlations of genome sizes with morphological traits were analyzed using ordinary least squares. A phylogenetically controlled analysis using the picante package [78] in R was also performed to fit a linear model to reveal the above relationships for the 112 species in the phylogenetic tree, which takes into account phylogenetic non-independence between data points. Data of spore size were only available for 102 moss species from relevant literature (Table S3); thus, we analyzed the relationships of spore size with genome size within these moss species.

## Data Availability

The data presented in this study are available from the corresponding author upon request.

## Supplementary Materials

The following supporting information can be downloaded at: https://www.mdpi.com/article/10.3390/plants12071564/s1, Table S1: Significance levels of difference among families in genome sizes (each with more than three species) ; Table S2: List of the 112 moss species including GenBank numbers for the four gene regions; Table S3: The data on plant size (at the individual level), leaf length and width (at the organ level), cell length and width, and spore diameter (at the cell level).

## Appendix A. Plant Materials, Voucher Number, Geographic Sources and DNA 1C Values (Means and Standard Errors)

**Families**	**Species**	**Localities**	**Lat** **(˚)**	**Lon** **(˚)**	**Ele** **(m)**	**Voucher Number**	**Genome Size Means** **(pg)**	**Genome Size SEs** **(pg)**
Sphagnaceae	*Sphagnum compactum* Lam. & DC.	Qixi Bridge, Mt. Niutou, Wuyi, Zhejiang Province	28.669	119.48	480	20170617098	0.504	0.023
Sphagnaceae	*Sphagnum recurvum* P. Beauv.	Meiduozegong, Jianziwan, Yajiang, Sichuan Province	29.348	100.113	4370	20170807033	0.526	0.014
Polytrichaceae	*Atrichum crispulum* Schimp. ex Besch. *	Laodian to Xianrending, Mt. Xitianmu, Linan, Zhejiang Province	30.342	119.428	1204	20170630022	0.59	0.006
Polytrichaceae	*Atrichum crispum* (James) Sull. *	Botanical Garden, Xuhui District, Shanghai Normal University	31.164	121.413	20	20170503003	0.561	0.006
Polytrichaceae	*Atrichum crispum* (James) Sull. *	near Jinhai Heavy Industry, Daishan, Zhejiang Province	30.244	122.263	33	1-20170530063	0.577	0.007
Polytrichaceae	*Pogonatum inflexum* (Lindb.) Sande Lac. *	Dachangtu, Daishan, Zhoushan District, Zhejiang Province	30.239	122.397	20	2-20170524037	0.603	0.019
Polytrichaceae	*Pogonatum inflexum* (Lindb.) Sande Lac. *	Mt. Foding, Mt. Putuo, Zhoushan District, Zhejiang Province	30.001	122.352	161	1-20170424282	0.601	0.008
Polytrichaceae	*Pogonatum nudiusculum* (Müll. Hal.) T.J. Kop. *	Camp 3 to Chengmen Cave, Hailuo Gully, Moxi Town, Luding County, Sichuan Province	29.576	102.001	2965	20170802052	0.508	0.004
Polytrichaceae	*Polytrichastrum emodi* G.L. Sm. **	Glaciers 1, Hailuo Gully, Moxi Town, Luding County, Sichuan Province	29.587	101.97	3505	20170801032	0.712	0.01
Polytrichaceae	*Polytrichastrum emodi* G.L. Sm. **	Glaciers 1, Hailuo Gully, Moxi Town, Luding County, Sichuan Province	29.587	101.97	3505	20170801034	0.533	0.012
Polytrichaceae	*Polytrichum commune* Hedw. *	Botanical Garden, Xuhui District, Shanghai Normal University	31.164	121.413	20	20170503002	0.474	0.005
Polytrichaceae	*Polytrichum juniperinum* Hedw.	Qixi Bridge, Mt. Niutou, Wuyi, Zhejiang Province	28.67	119.482	440	20170617081	0.76	0.019
Ptychomitriaceae ***	*Ptychomitrium dentatum* (Mitt.) A. Jaeger **	Upstairs, Mt. Xianren, Mt. Putuo, Zhoushan District, Zhejiang Province	30.028	121.906	229	20170221027	0.463	0.013
Ptychomitriaceae ***	*Ptychomitrium dentatum* (Mitt.) A. Jaeger **	Entrance, Hailuo Gully, Moxi Town, Luding County, Sichuan Province	29.651	102.115	1594	20170731042	0.573	0.004
Ptychomitriaceae ***	*Ptychomitrium linearifolium* Reim. & Sak. **	Roadside, Taizi Temple, Mt. Xitianmu, Linan, Zhejiang Province	30.325	119.45	408	20170627116	0.505	0.003
Ptychomitriaceae ***	*Ptychomitrium polyphylloides* (Müll. Hal.) Paris **	Camp 1, Hailuogou, Moxi Town, Luding County, Sichuan Province	29.603	102.074	1900	20170803182	0.544	0.003
Ptychomitriaceae ***	*Ptychomitrium tortula* (Harv.) A. Jaeger **	Camp 3 to Chengmen Cave, Hailuo Gully, Moxi Town, Luding County, Sichuan Province	29.573	102.003	2462	20170802118	0.496	0.007
Ptychomitriaceae ***	*Ptychomitrium wilsonii* Sull. & Lesq. **	Underbridge, Stele, Mt. Xitianmu, Linan, Zhejiang Province	30.323	119.447	337	20170627118	0.585	0.017
Grimmiaceae	*Grimmia pilifera* P. Beauv. **	Mt. Foding, Mt. Putuo, Zhoushan District, Zhejiang Province	29.992	122.383	97	1-20170424299	0.512	0.012
Grimmiaceae	*Grimmia pilifera* P. Beauv. **	Xianrending, Mt. Xitianmu, Linan, Zhejiang Province	30.35	119.424	1484	20170630052	0.591	0.015
Grimmiaceae	*Grimmia reflexidens* Müll. Hal. **	Roadside Daocheng Yading, Sichuan Province	28.764	100.277	4030	20170805060	0.488	0.005
Grimmiaceae	*Racomitrium anomodontoides* Cardot **	Tiesuo Bridge, Mt. Niutou, Wuyi, Zhejiang Province	28.671	119.481	570	20170617171	0.554	0.027
Grimmiaceae	*Racomitrium anomodontoides* Cardot **	Yixiantian, Mt. Niutou, Wuyi, Zhejiang Province	28.662	119.479	630	20170617148	0.578	0.012
Grimmiaceae	*Racomitrium anomodontoides* Cardot **	Laodian to Xianrending, Mt. Xitianmu, Linan, Zhejiang Province	30.342	119.428	1204	20170630009	0.563	0.012
Grimmiaceae	*Racomitrium anomodontoides* Cardot **	Simian Peak, Mt. Xitianmu, Linan, Zhejiang Province	30.341	119.434	1087	20170710141	0.516	0.037
Grimmiaceae	*Racomitrium ericoides* (Brid.) Brid. **	Dachangtu, Daishan, Zhoushan District, Zhejiang Province	30.264	122.36	45	2-20170529120	0.509	0.019
Grimmiaceae	*Racomitrium ericoides* (Brid.) Brid. **	Glaciers 1, Hailuo Gully, Moxi Town, Luding County, Sichuan Province	29.553	101.97	3505	20170801043	0.518	0.002
Grimmiaceae	*Racomitrium japonicum* Dozy & Molk. **	Fayu Temple to Fanyin Cave, Mt. Putuo, Zhoushan District, Zhejiang Province	30.004	122.401	56	2-20170424085	0.577	0.005
Grimmiaceae	*Schistidium striatum* Brid. *	Camp 3 to Chengmen Cave, Hailuo Gully, Moxi Town, Luding County, Sichuan Province	29.577	102.002	2983	20170802103	0.442	0.008
Grimmiaceae	*Schistidium subconfertum* (Broth.) Deguchi *	Downhill Telpher Station, Hailuo Gully, Moxi Town, Luding County, Sichuan Province	29.552	101.97	3557	20170801124	0.501	0.002
Dicranaceae	*Campylopus flexuosus* (Hedw.) Brid. **	Yixiantian, Mt. Niutou, Wuyi, Zhejiang Province	28.661	119.311	690	20170617148	0.567	0.014
Dicranaceae	*Campylopus umbellatus* (Arn.) Paris *	Dachangtu, Daishan, Zhoushan District, Zhejiang Province	30.239	122.397	45	20170529033	0.674	0.013
Dicranaceae	*Dicranodontium tenii* Broth. et Herz. *	Yixiantian, Mt. Niutou, Wuyi, Zhejiang Province	28.666	119.476	630	20170617127	0.653	0.021
Dicranaceae	*Dicranum gymnostomum* Mitt. *	Camp 3 to Chengmen Cave, Hailuo Gully, Moxi Town, Luding County, Sichuan Province	29.577	102.002	2983	20170802083	0.58	0.008
Dicranaceae	*Dicranum kashmirense* Broth. *	Qixi Bridge, Mt. Niutou, Wuyi, Zhejiang Province	28.668	119.481	480	20170617101	0.517	0.01
Dicranaceae	*Dicranum kashmirense* Broth. *	Xiagushanmen, Mt. Niutou, Wuyi, Zhejiang Province	28.671	119.485	450	20170617040	0.586	0.011
Dicranaceae	*Dicranum nipponense* Besch. *	Roadside from Laodian to Xianrending, Mt. Xitianmu, Linan, Zhejiang Province	30.342	119.399	1103	20170630025	0.608	0.006
Dicranaceae	*Oncophorus wahlenbergii* Brid. **	Camp 3 to Chengmen Cave, Hailuo Gully, Moxi Town, Luding County, Sichuan Province	29.571	101.998	2940	20170802150	0.62	0.005
Dicranaceae	*Oncophorus wahlenbergii* Brid. **	Camp 3 to Chengmen Cave, Hailuo Gully, Moxi Town, Luding County, Sichuan Province	29.576	102.072	2965	20170802028	0.508	0.008
Leucobryaceae ***	*Leucobryum bowringiii* Mitt. **	Xiagushanmen, Mt. Niutou, Wuyi, Zhejiang Province	28.674	119.5	420	20170617015	0.463	0.018
Leucobryaceae ***	*Leucobryum bowringiii* Mitt. **	Up Stairs, Chanyuan Temple, Mt. Xitianmu, Linan, Zhejiang Province	30.325	119.445	377	20170627113	0.596	0.002
Leucobryaceae ***	*Leucobryum glaucum* (Hedw.) Ångstr. **	Up Stairs, Chanyuan Temple, Mt. Xitianmu, Linan, Zhejiang Province	30.325	119.445	377	20170627113-1	0.458	0.019
Leucobryaceae ***	*Leucobryum juniperoideum* (Brid.) Müll. Hal. **	Xiaochangtu, Daishan, Zhoushan District, Zhejiang Province	30.244	122.262	43	1-2017053078	0.527	0.018
Leucobryaceae ***	*Leucobryum scabrum* Sande Lac. **	Xiagushanmen, Mt. Niutou, Wuyi, Zhejiang Province	28.671	119.485	450	20170617037	0.846	0.025
Leucobryaceae ***	*Paraleucobryum longifolium* (Ehrh. ex Hedw.) Loeske **	Glaciers 1, Hailuo Gully, Moxi Town, Luding County, Sichuan Province	29.587	101.97	3505	20170801061	0.655	0.019
Fissidentaceae	*Fissidens cristatus* Wilson ex Mitt. *	Qixi Bridge, Mt. Niutou, Wuyi, Zhejiang Province	28.67	119.472	460	20170617092	0.617	0.018
Fissidentaceae	*Fissidens cristatus* Wilson ex Mitt. *	Pavilion, Wannnian Temple, Mt. Emei Scenic Spot, Leshan District, Sichuan Province	29.593	103.376	810	20170810087	0.519	0.002
Fissidentaceae	*Fissidens dubius* P. Beauv.	Mt. Moxing, Daishan, Zhoushan District, Zhejiang Province	30.267	122.191	113	1-20170528023-2	0.607	0.018
Fissidentaceae	*Fissidens grandifrons* Brid. *	Caohaizi to Camp 2, Hailuo Gully, Moxi Town, Luding County, Sichuan Province	29.586	102.029	2663	20170803076	0.496	0.011
Fissidentaceae	*Fissidens teysmannianus* Dozy & Molk. *	Mt. Paotai, Daishan, Zhoushan District, Zhejiang Province	30.281	122.302	80	1-20170530033	0.581	0.019
Pottiaceae	*Barbula rigidula* (Hedw.) Mitt. **	Entrance, Hailuo Gully, Moxi Town, Luding County, Sichuan Province	29.649	102.115	1611	20170731002	0.66	0.015
Pottiaceae	*Hyophila propagulifera* Broth. **	Mt. Foding, Mt. Putuo, Zhoushan District, Zhejiang Province	30.012	122.384	303	1-20170424221	0.508	0.004
Pottiaceae	*Hyophila propagulifera* Broth. **	Farm Stay, Zhutuo Hill, Mt. Xitianmu, Linan, Zhejiang Province	30.328	119.454	453	20170627056	0.519	0.003
Pottiaceae	*Trichostomum hattorianum* B.C. Tan & Z. Iwats. *	Tianshi Peak, Mt. Niutou, Wuyi, Zhejiang Province	28.664	119.476	730	20170617158	0.597	0.017
Pottiaceae	*Weissia longifolia* DC. **	Roadside, Mt. Xianren, Mt. Putuo, Zhoushan District, Zhejiang Province	30.028	121.911	299	20170221113	0.532	0.012
Pottiaceae	*Weissia platyphylloides* Cardot **	Camp 3 to Dongga Temple, Hailuo Gully, Moxi Town, Luding County, Sichuan Province	29.568	101.993	2957	20170802201	0.492	0.002
Hedwigiaceae	*Hedwigia ciliata* (Hedw.) P. Beauv.	Guanyin Cave, Mt. Putuo, Zhoushan District, Zhejiang Province	29.985	122.373	124	2-20170426098	0.599	0.008
Hedwigiaceae	*Hedwigia ciliata* (Hedw.) P. Beauv.	Entrance, Bingchuanshihe Park, Mt. Haizi, Sichuan Province	29.296	100.082	4120	20170807001	0.48	0.004
Bartramiaceae ***	*Bartramia ithyphylla* Brid. **	Camp 3 to Chengmen Cave, Hailuo Gully, Moxi Town, Luding County, Sichuan Province	29.577	102.002	2983	20170802086	0.434	0.001
Bartramiaceae ***	*Philonotis marchica* (Hedw.) Brid. **	Gully, North of Fuyu Mountain Villa, Mt. Xitianmu, Linan, Zhejiang Province	30.322	119.448	340	20170627034	0.523	0.016
Bartramiaceae ***	*Philonotis mollis* (Dozy & Molk.) Mitt. **	Chanyuan Temple, Mt. Xitianmu, Linan, Zhejiang Province	30.323	119.442	390	20170629005	0.536	0.016
Bartramiaceae ***	*Philonotis revoluta* Bosch & Sande Lac. **	Caohaizi to Camp 2, Hailuo Gully, Moxi Town, Luding County, Sichuan Province	29.586	102.029	2663	20170803083	0.506	0.003
Bartramiaceae ***	*Philonotis revoluta* Bosch & Sande Lac. **	Tianshi Peak, Mt. Niutou, Wuyi, Zhejiang Province	28.664	119.476	730	20170617160	0.576	0.01
Bartramiaceae ***	*Philonotis thwaitesii* Mitt. **	Dachangtu, Daishan, Zhoushan District, Zhejiang Province	30.235	122.396	66	2-20170529053	0.542	0.012
Bryaceae	*Brachymenium nepalense* Hook. **	Xianrending, Mt. Xitianmu, Linan, Zhejiang Province	30.35	119.424	1484	20170630055	0.506	0.017
Bryaceae	*Brachymenium nepalense* Hook. **	Laba River, Mt. Erlang, Sichuan Province	29.981	102.443	1228	20170808054	0.531	0.013
Bryaceae	*Bryum argenteum* Hedw.	Shenniu Valley, Mt. Niutou, Wuyi, Zhejiang Province	28.666	119.787	70	20170617176	0.567	0.023
Bryaceae	*Bryum billarderi* Schwägr. *	Dachangtu, Daishan, Zhoushan District, Zhejiang Province	30.266	122.359	32	2-20170529118	0.686	0.004
Bryaceae	*Bryum billarderii* Schwägr. *	Laodian to Xianrending, Mt. Xitianmu, Linan, Zhejiang Province	30.342	119.428	1204	20170630026	0.596	0.015
Bryaceae	*Pohlia cruda* (Hedw.) Lindb. *	Glaciers 1, Hailuo Gully, Moxi Town, Luding County, Sichuan Province	29.552	101.97	3554	20170801087	0.471	0.005
Bryaceae	*Pohlia timmioides* Broth. *	Glaciers 1, Hailuo Gully, Moxi Town, Luding County, Sichuan Province	29.553	101.969	3541	20170801074	0.661	0.004
Bryaceae	*Rhodobryum giganteum* (Schwägr.) Paris *	Yixiantian, Mt. Niutou, Wuyi, Zhejiang Province	28.662	119.478	660	20170617140	0.862	0.006
Mniaceae	*Mnium spinosum* (Voit) Schwägr. *	Downhill Telpher Station to Heisonglin, Moxi Town, Luding County, Sichuan Province	29.574	101.993	3030	20170801160	0.725	0.013
Mniaceae	*Plagiomnium acutum* (Lindb.) T.J. Kop. *	Botanical Garden, Xuhui District, Shanghai Normal University	31.164	121.413	20	20170503004	0.561	0.006
Mniaceae	*Plagiomnium acutum* (Lindb.) T.J. Kop. *	near Jinhai Heavy Industry, Daishan, Zhejiang Province	30.244	122.263	33	1-20170530065	0.536	0.013
Mniaceae	*Plagiomnium arbusculum* (Müll. Hal.) T.J. Kop. *	Camp 3 to Chengmen Cave, Hailuo Gully, Moxi Town, Luding County, Sichuan Province	29.577	102.002	2983	20170802099	0.526	0.002
Mniaceae	*Plagiomnium maximovicgii* (Lindb) T. Kop. *	Qixi Bridge, Mt. Niutou, Wuyi, Zhejiang Province	28.666	119.476	480	20170617114	0.599	0.019
Mniaceae	*Plagiomnium maximovicgii* (Lindb) T. Kop. *	Gully, North of Fuyu Mountain Villa, Mt. Xitianmu, Linan, Zhejiang Province	30.322	119.448	340	20170629053	0.64	0.02
Mniaceae	*Plagiomnium maximovicgiiv* (Lindb) T. Kop. *	Downstairs, Liuchun House, Mt. Xitianmu, Linan, Zhejiang Province	30.322	119.446	369	20170627138	0.657	0.029
Mniaceae	*Rhizomnium gracile* T.J. Kop. *	Camp 3 to Dongga Temple, Hailuo Gully, Moxi Town, Luding County, Sichuan Province	29.568	101.993	2957	20170802195	0.558	0.012
Mniaceae	*Trachycystis microphylla* (Dozy & Molk.) Lindb. *	near Jinhai Heavy Industry, Daishan, Zhejiang Province	30.244	122.263	33	1-20170530062	0.619	0.01
Mniaceae	*Trachycystis microphylla* (Dozy & Molk.) Lindb. **	Botanical Garden, Xuhui District, Shanghai Normal University	31.164	121.413	20	20170503001	0.528	0.016
Orthotrichaceae	*Macromitrium gymnostomum* Sull. & Lesq. **	Jiufeng Park, Taizhou, Zhejiang Pronvice	28.906	112.464	45	20160619011	0.459	0.023
Orthotrichaceae	*Macromitrium gymnostomum* Sull. & Lesq. **	Tiesuo Bridge, Mt. Niutou, Wuyi, Zhejiang Province	28.664	119.478	510	20170617121	0.572	0.014
Orthotrichaceae	*Macromitrium japonicum* Dozy & Molk. **	Roadside of Fushan Temple, Shengsi, Zhoushan, Zhejiang Province	30.725	122.821	20	20160405204	0.422	0.007
Orthotrichaceae	*Orthotrichum brassii* E.B. Bartram *	Camp 3 to Chengmen Cave, Hailuo Gully, Moxi Town, Luding County, Sichuan Province	29.57	101.998	2930	20170802175	0.602	0.013
Orthotrichaceae	*Orthotrichum erubescens* Müll. Hal. *	Fayu Temple to Fanyin Cave, Mt. Putuo, Zhoushan District, Zhejiang Province	30.004	122.403	40	2-20170424092	0.504	0.007
Orthotrichaceae	*Orthotrichum hookeri* Wilson ex Mitt. *	Camp 3 to Chengmen Cave, Hailuo Gully, Moxi Town, Luding County, Sichuan Province	29.573	102.003	2927	20170802137	0.528	0.003
Orthotrichaceae	*Schlotheimia pungens* E.B. Bartram **	Tianshi Peak, Mt. Niutou, Wuyi, Zhejiang Province	28.664	119.476	730	20170617159	0.558	0.025
Orthotrichaceae	*Ulota crispa* (Hedw.) Brid. **	Heisonglin, Hailuo Gully, Moxi Town, Luding County, Sichuan Province	29.574	101.991	3043	20170801164	0.524	0.005
Aulacomniaceae	*Aulacomnium heterostichum* (Hedw.) Bruch & Schimp.	Jieyin Temple to Leidongping, Mt. Emei Scenic Spot, Leshan District, Sichuan Province	29.536	103.327	2452	20170810040	0.51	0.005
Rhizogoniaceae ***	*Pyrrhobryum dozyanum* (Sande Lac.) Manuel **	Yixiantian, Mt. Niutou, Wuyi, Zhejiang Province	28.666	119.476	640	20170617133	0.5	0.014
Rhizogoniaceae ***	*Pyrrhobryum spiniforme* (Hedw.) Mitt. **	Tianshi Palace, Mt. Niutou, Wuyi, Zhejiang Province	28.666	119.476	480	20170617115	0.572	0.016
Racopilaceae ***	*Racopilum cuspidigerum* (Schwägr.) Ångstr. **	Downstairs, Liuchun House, Mt. Xitianmu, Linan, Zhejiang Province	30.322	119.446	369	20170627135	0.478	0.021
Hypopterygiaceae ***	*Hypopterygium japonicum* Mitt. **	Gully, Fuyu Mountain Villa to Zhonglie Temple, Mt. Xitianmu, Linan, Zhejiang Province	30.323	119.447	385	20170629011	0.505	0.006
Hookeriaceae	*Hookeria acutifolia* Hook. & Grev. *	Water side, Mt. Putuo, Zhoushan District, Zhejiang Province	30.088	122.509	106	1-20170216272	0.458	0.018
Hookeriaceae	*Hookeria acutifolia* Hook. & Grev. *	near Laodian, Mt. Xitianmu, Linan, Zhejiang Province	30.342	119.435	1129	20170630065	0.497	0.016
Plagiotheciaceae	*Plagiothecium handelii* Broth. *	Camp 3 to Chengmen Cave, Hailuo Gully, Moxi Town, Luding County, Sichuan Province	29.571	101.914	2940	20170802157	0.491	0.006
Plagiotheciaceae	*Plagiothecium nemorale* (Mitt.) A. Jaeger *	Huiji Temple, Mt. Putuo, Zhoushan District, Zhejiang Province	30.013	122.352	290	1-20170424188	0.545	0.007
Plagiotheciaceae	*Plagiothecium nemorale* (Mitt.) A. Jaeger *	Tianshi Palace, Mt. Niutou, Wuyi, Zhejiang Province	28.67	119.472	460	20170617094	0.562	0.01
Plagiotheciaceae	*Plagiothecium nemorale* (Mitt.) A. Jaeger *	Laodian, Mt. Xitianmu, Linan, Zhejiang Province	30.342	119.434	1099	20170710054	0.536	0.016
Plagiotheciaceae	*Plagiothecium piliferum* (Sw.) Schimp. *	West of Hong Temple, Mt. Xitianmu, Linan, Zhejiang Province	30.324	119.439	447	20170627020	0.581	0.012
Climaciaceae	*Climacium dendroides* (Hedw.) F. Weber & D. Mohr *	Laodian to Xianrending, Mt. Xitianmu, Linan, Zhejiang Province	30.342	119.433	1098	20170630006	0.571	0.01
Climaciaceae	*Climacium dendroides* (Hedw.) F. Weber & D. Mohr *	Glaciers 1, Hailuo Gully, Moxi Town, Luding County, Sichuan Province	29.587	101.97	3505	20170801031	0.737	0.003
Climaciaceae	*Climacium dendroides* (Hedw.) F. Weber & D. Mohr *	Hailuo Gully, Moxi Town, Luding County, Sichuan Province	29.552	101.97	3538	20170801012	0.626	0.017
Amblystegiaceae	*Calliergon cordifolium* (Hedw.) Kindb. *	Gully, Fuyu Mountain Villa to Zhonglie Temple, Mt. Xitianmu, Linan, Zhejiang Province	30.323	119.447	385	20170629048	0.56	0.016
Amblystegiaceae	*Cratoneuron filicinum* (Hedw.) Spruce	Kangding, Sichuan Province	30.009	101.952	2812	20170808039	0.463	0.003
Amblystegiaceae	*Sanionia uncinata* (Hedw.) Loeske **	Glaciers 1, Hailuo Gully, Moxi Town, Luding County, Sichuan Province	29.552	101.97	3554	20170801087	0.498	0.011
Amblystegiaceae	*Sanionia uncinata* (Hedw.) Loeske **	Camp 3 to Chengmen Cave, Hailuo Gully, Moxi Town, Luding County, Sichuan Province	29.573	102.003	2462	20170802134	0.581	0.004
Leskeaceae	*Lescuraea radicosa* (Mitt.) Mönk. **	Camp 3 to Chengmen Cave, Hailuo Gully, Moxi Town, Luding County, Sichuan Province	29.577	102.001	2946	20170802016	0.788	0.011
Thuidiaceae	*Actinothuidium hookeri* (Mitt.) Broth. **	Hailuo Gully, Moxi Town, Luding County, Sichuan Province	29.574	101.993	3010	20170801143	0.502	0.003
Thuidiaceae	*Actinothuidium hookeri* (Mitt.) Broth. **	Downhill Telpher Station, Hailuo Gully, Moxi Town, Luding County, Sichuan Province	29.552	101.97	3557	20170801132	0.669	0.027
Thuidiaceae	*Anomodon viticulosus* (Hedw.) Hook. & Taylor	Liuchun House, Mt. Xitianmu, Linan, Zhejiang Province	30.322	119.445	377	20170627092	0.534	0.017
Thuidiaceae	*Boulaya mittenii* (Broth.) Cardot **	Camp 3 to Chengmen Cave, Hailuo Gully, Moxi Town, Luding County, Sichuan Province	29.576	102.001	2965	20170802044	0.612	0.003
Thuidiaceae	*Cyrtohypnum bonianum* (Besch.) W.R. Buck & H.A. Crum **	Xiagushanmen, Mt. Niutou, Wuyi, Zhejiang Province	28.674	119.5	430	20170617026	0.671	0.01
Thuidiaceae	*Cyrtohypnum pygmaeum* (Schimp.) Buck et Crum **	Botanical Garden, Xuhui District, Shanghai Normal University	31.164	121.413	20	20170509002	0.541	0.011
Thuidiaceae	*Haplocladium angustifolium* (Hampe & Müll. Hal.) Broth. *	Botanical Garden, Xuhui District, Shanghai Normal University	31.164	121.413	20	20170509001	0.51	0.019
Thuidiaceae	*Haplohymenium triste* (Ces.) Kindb. *	Liuchun House, Mt. Xitianmu, Linan, Zhejiang Province	30.322	119.445	377	20170629027	0.528	0.012
Thuidiaceae	*Herpetineuron toccoae* (Sull. & Lesq.) Cardot **	Dachangtu, Daishan, Zhoushan District, Zhejiang Province	30.261	122.341	117	1-20170530013	0.619	0.025
Thuidiaceae	*Herpetineuron toccoae* (Sull. & Lesq.) Cardot **	Pavilion, Wannnian Temple, Mt. Emei Scenic Spot, Leshan District, Sichuan Province	29.643	103.476	816	20170810082	0.571	0.003
Thuidiaceae	*Pinnatella anacamptolepis* (Müll. Hal.) Broth. **	Qixi Bridge, Mt. Niutou, Wuyi, Zhejiang Province	28.668	119.472	470	20170617097	0.545	0.014
Thuidiaceae	*Thuidium cymbifolium* (Dozy & Molk.) Dozy & Molk. *	Entrance, Hailuo Gully, Moxi Town, Luding County, Sichuan Province	29.649	102.115	1611	20170731018	0.599	0.015
Thuidiaceae	*Thuidium kanedae* Sakurai *	Roadside, Taizi Temple, Mt. Xitianmu, Linan, Zhejiang Province	30.324	119.439	447	20170627115	0.659	0.021
Brachytheciaceae	*Brachythecium buchananii* (Hook.) A. Jaeger *	Entrance, Hailuo Gully, Moxi Town, Luding County, Sichuan Province	29.649	102.115	1611	20170731013	0.554	0.002
Brachytheciaceae	*Brachythecium piligerum* Cardot *	Jieyin Temple to Leidongping, Mt. Emei Scenic Spot, Leshan District, Sichuan Province	29.502	103.331	2091	20170810021	0.547	0.005
Brachytheciaceae	*Brachythecium plumosum* (Hedw.) Schimp. *	Botanical Garden, Xuhui District, Shanghai Normal University	31.164	121.413	20	20170517001	0.492	0.013
Brachytheciaceae	*Cirriphyllum cirrosum* (Schwägr.) Grout *	Camp 3 to Chengmen Cave, Hailuo Gully, Moxi Town, Luding County, Sichuan Province	29.577	102.001	2946	20170802014	0.606	0.003
Brachytheciaceae	*Eurhynchium hians* (Hedw.) Sande Lac.	Lianfeng Road, Mt. Xitianmu, Linan, Zhejiang Province	30.341	119.436	1101	20170710117	0.498	0.008
Brachytheciaceae	*Eurhynchium savatieri* Schimp. ex Besch. *	Mt. Moxing, Daishan, Zhoushan District, Zhejiang Province	30.264	122.192	180	1-20170528006-2	0.589	0.019
Brachytheciaceae	*Myuroclada maximowiczii* (G.G. Borshch.) Steere & W.B. Schofield **	Mt. Moxing, Daishan, Zhoushan District, Zhejiang Province	30.269	122.36	68	1-20170528032-3	0.498	0.015
Brachytheciaceae	*Palamocladium leskeoides* (Hook.) E. Britton **	Jinshanmen, Mt. Xitianmu, Linan, Zhejiang Province	30.326	119.441	341	20170629012	0.54	0.008
Brachytheciaceae	*Rhynchostegium pallidifolium* (Mitt.) A. Jaeger *	Dachangtu, Daishan, Zhoushan District, Zhejiang Province	30.239	122.397	13	2-20170529046	0.579	0.025
Brachytheciaceae	*Rhynchostegium riparioides* (Hedw.) Cardot *	Mt. Moxing, Daishan, Zhoushan District, Zhejiang Province	30.266	122.191	143	1-20170528019-2	0.564	0.017
Hypnaceae	*Homomallium connexum* (Cardot) Broth. *	Telpher Station, Mt. Putuo, Zhoushan District, Zhejiang Province	30.026	122.397	71	1-20170425044	0.458	0.016
Hypnaceae	*Homomallium connexum* (Cardot) Broth. *	Farm Stay, Zhutuo Hill, Mt. Xitianmu, Linan, Zhejiang Province	30.328	119.454	453	20170627052	0.507	0.017
Hypnaceae	*Homomallium connexum* (Cardot) Broth. *	Mali Tree, Liuchun House, Mt. Xitianmu, Linan, Zhejiang Province	30.322	119.445	377	20170629045	0.572	0.027
Hypnaceae	*Hypnum cupressiforme* Hedw. *	Dachangtu, Daishan, Zhoushan District, Zhejiang Province	30.235	122.396	66	2-20170529049	0.465	0.011
Hypnaceae	*Hypnum plumaeforme* Wilson *	Botanical Garden, Xuhui District, Shanghai Normal University	31.164	121.413	20	20170509003	0.48	0.022
Hypnaceae	*Hypnum revolutum* (Mitt.) Lindb. *	Entrance, Bingchuanshihe Park, Mt. Haizi, Sichuan Province	29.298	100.082	4140	20170807012	0.593	0.013
Hypnaceae	*Pseudotaxiphyllum pohliaecarpum* (Sull. & Lesq.) Z. Iwats. **	Mt. Mahuang, Mt. Putuo, Zhoushan District, Zhejiang Province	30.062	122.052	109	2-20170430033	0.617	0.003
Hypnaceae	*Pseudotaxiphyllum pohliaecarpum* (Sull. & Lesq.) Z. Iwats. **	Qixi Bridge, Mt. Niutou, Wuyi, Zhejiang Province	28.671	119.484	460	20170617050	0.578	0.021
Hypnaceae	*Ptilium crista-castrensis* (Hedw.) De Not. **	Hailuo Gully, Moxi Town, Luding County, Sichuan Province	29.651	102.115	1594	20170801007	0.571	0.025
Hypnaceae	*Ptilium crista-castrensis* (Hedw.) De Not. **	Glaciers 1, Hailuo Gully, Moxi Town, Luding County, Sichuan Province	29.553	101.969	3541	20170801067	0.659	0.011
Hypnaceae	*Taxiphyllum taxirameum* (Mitt.) M. Fleisch. *	Tianshi Palace, Mt. Niutou, Wuyi, Zhejiang Province	28.666	119.476	480	20170617116	0.536	0.024
Meteoriaceae	*Aerobryidium filamentosum* (Hook.) M. Fleisch. **	Qixi Bridge, Mt. Niutou, Wuyi, Zhejiang Province	28.673	119.485	420	20170617075	0.589	0.018
Meteoriaceae	*Chrysocladium retrorsum* (Mitt.) M. Fleisch. **	Qixi Bridge, Mt. Niutou, Wuyi, Zhejiang Province	28.668	119.481	480	20170617107	0.642	0.003
Meteoriaceae	*Meteorium subpolytrichum* (Besch.) Broth. *	Yixiantian, Mt. Niutou, Wuyi, Zhejiang Province	28.663	119.312	670	20170617161	0.535	0.008
Meteoriaceae	*Neodicladiella pendula* (Sull.) W.R. Buck **	Yixiantian, Mt. Niutou, Wuyi, Zhejiang Province	28.666	119.476	490	20170617112	0.598	0.023
Meteoriaceae	*Neodicladiella pendula* (Sull.) W.R. Buck **	Camp 3 to Chengmen Cave, Hailuo Gully, Moxi, Luding County, Sichuan Province	29.576	102.072	2965	20170802030	0.654	0.01
Meteoriaceae	*Neodicladiella pendula* (Sull.) W.R. Buck **	Camp 3 to Chengmen Cave, Hailuo Gully, Moxi, Luding County, Sichuan Province	29.573	102.003	2462	20170802118	0.576	0.018
Sematophyllaceae	*Barbella spiculata* (Mitt.) Broth. **	Downhill Telpher Station to Heisonglin, Hailuo Gully, Moxi, Luding County, Sichuan Province	29.575	101.993	2997	20170801152	0.482	0.001
Sematophyllaceae	*Brotherella falcata* (Dozy & Molk.) M. Fleisch. **	Downhill Telpher Station, Hailuo Gully, Moxi, Luding County, Sichuan Province	29.574	101.993	3010	20170801137	0.565	0.005
Sematophyllaceae	*Sematophyllum subhumile* (Müll. Hal.) M. Fleisch. **	Mt. Moxing, Daishan, Zhoushan District, Zhejiang Province	30.266	122.191	120	2-20170726015	0.557	0.01
Sematophyllaceae	*Trichosteleum lutschianum* (Broth. & Paris) Broth. **	Yixiantian, Mt. Niutou, Wuyi, Zhejiang Province	28.666	119.476	630	20170617130	0.498	0.013
Trachypodaceae ***	*Trachypus bicolor* Reinw. & Hornsch. *	Laodian to Xianrending, Mt. Xitianmu, Linan, Zhejiang Province	30.342	119.428	1204	20170630039	0.571	0.007
Hylocomiaceae	*Hylocomiastrum himalayanum* (Mitt.) Broth. **	Glaciers 1, Hailuo Gully, Moxi Town, Luding County, Sichuan Province	29.553	101.969	3541	20170801064	0.578	0.008
Hylocomiaceae	*Hylocomium splendens* (Hedw.) Schimp.	Downhill Telpher Station, Hailuo Gully, Moxi, Luding County, Sichuan Province	29.552	101.97	3557	20170801133	0.548	0.012
Hylocomiaceae	*Hylocomium splendens* (Hedw.) Schimp.	Camp 3 to Chengmen Cave, Hailuo Gully, Moxi, Luding County, Sichuan Province	29.571	101.914	2940	20170802160	0.644	0.01
Rhytidiaceae	*Rhytidiadelphus triquetrus* (Hedw.) Warnst.	Glaciers 1, Hailuo Gully, Moxi Town, Luding County, Sichuan Province	29.552	101.97	3557	20170801091	0.81	0.022
Rhytidiaceae	*Rhytidiadelphus triquetrus* (Hedw.) Warnst.	Camp 3 to Chengmen Cave, Hailuo Gully, Moxi Town, Luding County, Sichuan Province	29.57	101.998	2930	20170802176	0.749	0.011
Rhytidiaceae	*Rhytidium rugosum* (Hedw.) Kindb.	Entrance, Bingchuanshihe Park, Mt. Haizi, Sichuan Province	29.298	100.082	4140	20170807009	0.509	0.012
Rhytidiaceae	*Rhytidium rugosum* (Hedw.) Kindb.	Foot of Mt. Bowa, Daocheng, Sichuan Province	29.593	100.289	4080	20170805017	0.563	0.016
Entodontaceae	*Entodon concinnus* (De Not.) Paris *	Shenniu Valley, Mt. Niutou, Wuyi, Zhejiang Province	28.956	119.794	70	20170617173	0.496	0.023
Entodontaceae	*Entodon divergens* Broth. *	Qixi Bridge, Mt. Niutou, Wuyi, Zhejiang Province	28.668	119.481	480	20170617106	0.544	0.012
Entodontaceae	*Entodon macropodus* (Hedw.) Müll. Hal. *	Qixi Bridge, Mt. Niutou, Wuyi, Zhejiang Province	28.671	119.484	460	20170617055	0.522	0.005
Entodontaceae	*Entodon prorepens* (Mitt.) A. Jaeger *	Entrance, Hailuo Gully, Moxi Town, Luding County, Sichuan Province	29.65	102.115	1611	20170731023	0.459	0.006
Entodontaceae	*Entodon sullivantii* (Müll. Hal.) Lindb. *	Yinxiu Nunnery, Mt. Putuo, Zhoushan District, Zhejiang Province	29.978	122.377	37	2-20170426064	0.528	0.01
Entodontaceae	*Pleurozium schreberi* (Brid.) Mitt.	Yixiantian, Mt. Niutou, Wuyi, Zhejiang Province	28.666	119.476	650	20170617135	0.549	0.008
Entodontaceae	*Pleurozium schreberi* (Brid.) Mitt.	Xianrending, Mt. Xitianmu, Linan, Zhejiang Province	30.35	119.424	1484	20170630049	0.625	0.012
Entodontaceae	*Pleurozium schreberi* (Brid.) Mitt.	Camp 3 to Chengmen Cave, Hailuo Gully, Moxi Town, Luding County, Sichuan Province	29.571	101.914	2940	20170802161	0.469	0.008
Leucodontaceae	*Dozya japonica* Sande Lac. **	Farm Stay, Linzhongyuan, Mt. Xitianmu, Linan, Zhejiang Province	30.328	119.454	452	20170627063	0.567	0.025
Leucodontaceae	*Leucodon secundus* (Harv.) Mitt. **	Hailuo Gully, Moxi Town, Luding County, Sichuan Province	29.575	101.993	2997	20170801150	0.516	0.015
Leucodontaceae	*Leucodon secundus* (Harv.) Mitt. **	Camp 3 to Chengmen Cave, Hailuo Gully, Moxi Town, Luding County, Sichuan Province	29.571	101.914	2940	20170802155	0.496	0.002
Leucodontaceae	*Leucodon subulatulus* Broth. **	Camp 3 to Chengmen Cave, Hailuo Gully, Moxi Town, Luding County, Sichuan Province	29.574	101.992	3020	20170802003	0.548	0.01
Cryphaeaceae ***	*Pilotrichopsis robusta* P.C. Chen **	Camp 3 to Chengmen Cave, Hailuo Gully, Moxi Town, Luding County, Sichuan Province	29.571	101.914	2940	20170802163	0.472	0.011
Neckeraceae	*Homalia trichomanoides* (Hedw.) Schimp. *	Gully, Fuyu Mountain Villa to Zhonglie Temple, Mt. Xitianmu, Linan, Zhejiang Province	30.323	119.447	385	20170629054	0.522	0.013
Neckeraceae	*Homalia trichomanoides* (Hedw.) Schimp. *	Mt. Wutong Scenic Spot, Shenzhen	22.564	114.222	160	20170726003	0.716	0.007
Neckeraceae	*Homaliodendron flabellatum* (Sm.) M. Fleisch. *	Tianshi Palace, Mt. Niutou, Wuyi, Zhejiang Province	28.668	119.481	480	20170617104	0.542	0.008
Neckeraceae	*Homaliodendron ligulaefolium* (Mitt.) M. Fleisch. *	Laodian to Xianrending, Mt. Xitianmu, Linan, Zhejiang Province	30.342	119.428	1204	20170630011	0.601	0.017
Neckeraceae	*Homaliodendron scalpellifolium* (Mitt.) M. Fleisch. *	Yixiantian, Mt. Niutou, Wuyi, Zhejiang Province	28.666	119.476	650	20170617136	0.558	0.013
Neckeraceae	*Homaliodendron scalpellifolium* (Mitt.) M. Fleisch. *	Camp 3 to Dongga Temple, Hailuo Gully, Moxi Town, Luding County, Sichuan Province	29.568	101.993	2957	20170802202	0.492	0.013
Neckeraceae	*Neckera decurrens* Broth. *	Hailuo Gully, Moxi Town, Luding County, Sichuan Province	29.651	102.115	1594	20170801004	0.493	0.016
Neckeraceae	*Neckera humilis* Mitt. *	Huiji Temple, Mt. Putuo, Zhoushan District, Zhejiang Province	30.013	122.385	236	1-20170424174	0.51	0.007
Neckeraceae	*Neckera humilis* Mitt. *	Huiji Temple, Mt. Putuo, Zhoushan District, Zhejiang Province	30.013	122.385	236	1-20170424174	0.587	0.003
Neckeraceae	*Neckera yezoana* Besch. *	Pavilion, Wannnian Temple, Mt. Emei Scenic Spot, Leshan District, Sichuan Province	29.593	103.376	810	20170810092	0.595	0.006
Neckeraceae	*Thamnobryum subseriatum* (Mitt. ex Sande Lac.) B.C. Tan *	Huiji Temple, Mt. Putuo, Zhoushan District, Zhejiang Province	30.013	122.352	290	1-20170424184	0.587	0.013
Neckeraceae	*Thamnobryum subseriatum* (Mitt. ex Sande Lac.) B.C. Tan *	Dashuwang, Mt. Xitianmu, Linan, Zhejiang Province	30.341	119.433	1075	20170710065	0.532	0.038
Neckeraceae	*Thamnobryum subseriatum* (Sw.) Schimp. *	Gully, Left Hand of Stele, Fuyu Mountain Villa, Mt. Xitianmu, Linan, Zhejiang Province	30.322	119.448	340	20170629056	0.497	0.007
Marchantiaceae	*Marchantia polymorpha* L.	Shenniu Valley, Mt. Niutou, Wuyi, Zhejiang Province	28.666	119.787	70	20170617181	0.518	0.018
Marchantiaceae	*Marchantia polymorpha* L.	Qixi Bridge, Mt. Niutou, Wuyi, Zhejiang Province	28.666	119.476	480	20170617118	0.552	0.027
Dumortieraceae ***	*Dumortiera hirsuta* (Sw.) Nees **	Tiesuo Bridge, Mt. Niutou, Wuyi, Zhejiang Province	28.666	119.476	660	20170617138	0.552	0.03
Dumortieraceae ***	*Dumortiera hirsuta* (Sw.) Nees **	Roadside, Fuyu Mountain Villa to Zhonglie Temple, Mt. Xitianmu, Linan, Zhejiang Province	30.323	119.447	376	20170627007	0.621	0.019
Pallaviciniaceae	*Pallavicinia ambigua* (Mitt.) Steph. *	Qixi Bridge, Mt. Niutou, Wuyi, Zhejiang Province	28.67	119.481	410	20170617088	0.52	0.005
Pallaviciniaceae	*Pallavicinia ambigua* (Mitt.) Steph. *	Qixi Bridge, Mt. Niutou, Wuyi, Zhejiang Province	28.668	119.481	480	20170617102	0.534	0.005
Cephaloziaceae	*Alobiellopsis parvifolius* (Steph.) R. M. Schust. **	Water side, Mt. Putuo, Zhoushan District, Zhejiang Province	30.028	121.906	229	20170221042	0.471	0.015
Scapaniaceae	*Scapania verrucosa* Heeg *	Xiagushanmen, Mt. Niutou, Wuyi, Zhejiang Province	28.671	119.484	460	20170617048	0.496	0.004
Trichocoleaceae	*Trichocolea tomentella* (Ehrh.) Dumort.	Camp 3 to Dongga Temple, Hailuo Gully, Moxi Town, Luding County, Sichuan Province	29.568	101.993	2957	20170802193	0.653	0.004
Lepidoziaceae	*Bazzania denudata* (Torr. ex Gottsche, Lindenb. & Nees) Trevis.	Xiagushanmen, Mt. Niutou, Wuyi, Zhejiang Province	28.673	119.485	410	20170617007	0.641	0.028
Lepidoziaceae	*Bazzania praerupta* (Reinw.Blume & Nees) Trevis. *	Xiagushanmen, Mt. Niutou, Wuyi, Zhejiang Province	28.674	119.5	430	20170617018	0.551	0.006
Lepidoziaceae	*Lepidozia subintegra* Lindenb. *	Tiesuo Bridge, Mt. Niutou, Wuyi, Zhejiang Province	28.666	119.476	640	20170617132	0.514	0.017
Plagiochilaceae	*Plagiochila chinensis* Steph. *	Camp 3 to Dongga Temple, Hailuo Gully, Moxi Town, Luding County, Sichuan Province	29.568	101.993	2957	20170802191	0.673	0.007
Plagiochilaceae	*Plagiochila durelii* Schiffn. *	Downstairs, Liuchun House, Mt. Xitianmu, Linan, Zhejiang Province	30.322	119.446	369	20170629036	0.542	0.018
Plagiochilaceae	*Plagiochila griffithiana* Steph. *	Yixiantian, Mt. Niutou, Wuyi, Zhejiang Province	28.666	119.476	640	20170617131	0.614	0.003
Plagiochilaceae	*Plagiochila zonata* Steph. *	Camp 3 to Dongga Temple, Hailuo Gully, Moxi Town, Luding County, Sichuan Province	29.568	101.993	2957	20170802190	0.53	0.012
Porellaceae	*Porella caespitans* (Steph.) S. Hatt. *	Hailuo Gully, Moxi Town, Luding County, Sichuan Province	29.575	101.993	2997	20170801154	0.612	0.007
Porellaceae	*Porella chinensis* (Steph.) Hatt. *	Liuchun House, Mt. Xitianmu, Linan, Zhejiang Province	30.323	119.445	376	20170627130	0.613	0.013
Frullaniaceae	*Frullania ericoides* (Nees) Mont. *	Lingshi Temple, Mt. Putuo, Zhoushan District, Zhejiang Province	29.986	122.372	121	3-20170425108	0.531	0.005
Lejeuneaceae	*Trocholejeunea sandvicensis* (Gottsche) Mizut. **	Fuyu Mountain Villa to Zhonglie Temple, Mt. Xitianmu, Linan, Zhejiang Province	30.323	119.447	376	20170627045	0.659	0.021
Metzgeriaceae	*Metzgeria novicrassipilis* Kuwah. *	Qixi Bridge, Mt. Niutou, Wuyi, Zhejiang Province	28.67	119.482	440	20170617080	0.664	0.004

Note: Newly reported families, genera and species are marked with three asterisks, two asterisks, and one asterisk, respectively.
