# Supplementary material for: Nuclear DNA Amounts in Chinese Bryophytes Estimated by Flow Cytometry: Variation Patterns and Biological Significances
Source: Plants (Basel). 2023 Apr 5;12(7):1564. doi: 10.3390/plants12071564 (PMC10096954; doi:10.3390/plants12071564)
Supplement: Supplementary file 1 [file plants-12-01564-s001.zip › Table S1.pdf]

Table S1. Significance levels of difference among families in genome sizes (each with more than three species)

	Leucobryaceae	Rhytidiaceae	Entodontaceae	Orthotrichaceae	Dicranaceae	Mniaceae
Rhytidiaceae	N					
Entodontaceae	0.033	0.021				
Orthotrichaceae	0.039	0.025	N			
Dicranaceae	N	N	0.022	0.026		
Mniaceae	N	N	0.046	N	N	
Bartramiaceae	N	0.048	N	N	N	N
Grimmiaceae	0.037	0.021	N	N	0.02	0.044

N：P > 0.05
